# Supplementary material for: Allergies, Allergic Comorbidities and the Home Environment in Pediatric Asthma in Southern Florida
Source: Int J Environ Res Public Health. 2021 Apr 14;18(8):4142. doi: 10.3390/ijerph18084142 (PMC8070846; doi:10.3390/ijerph18084142)
Supplement: Supplementary file 1 [file ijerph-18-04142-s001.pdf]

SUPPLEMENT ONLINE MATERIAL

Allergies, Allergic Comorbidities and the Home Environment in Pediatric Asthma in Southern Florida - Supplement Online Material

Nadia T. Saif [1], Gary I. Kleiner [2], Lourdes Q. Forster [2], Eugene R. Hershorin [2], Andrew A. Colin[3], Mehdi Mirsaeidi [4] and Naresh Kumar [1,*]

[1] Department of Public Health Sciences, University of Miami Miller School of Medicine, Miami, FL 33136, USA; nadia.thura@gmail.com (N.T.S.)
[2] Department of Pediatrics, University of Miami Health System, Miami, FL 33136, USA; gary.kleiner@med.miami.edu (G.I.K.); lforster@med.miami.edu (L.Q.F.); ehershorin@med.miami.edu (E.R.H.)
[3] , Division of Pediatric Pulmonology, Department of Pediatrics, University of Miami Miller School of Medicine, Miami, FL 33136, USA; acolin@med.miami.edu
[4] Pulmonary, Allergy, Critical Care and Sleep Medicine Division, University of Miami Miller School of Medicine, Miami, FL 33136, USA , msm249@med.miami.edu
* Correspondence: nkumar@miami.edu; Tel: +1-305-243-4854

Survey Instrument

1. Date of birth (dd/mm/yyyy): **<u>dd/mm/yyyy</u>**

2. Gender: Female ☐ Male ☐

3. Where was your child born?
   - o Florida ☐
   - o Outside Florida, but in U.S. ☐
     - o When did your child move to Florida (month and year)? **<u>mm/yyyy</u>**
   - o Outside U.S. ☐
     - o When did your child move to Florida (month and year)? **<u>mm/yyyy</u>**

4. Birth place: **name of birth city:______ state:____ 5-digit zip code:_____**
OR, if abroad, country: ______

4. Current residential address: **city, zip code:________________**

5. Does anyone who lives in your child's home smoke?
   - Yes ☐
   - No ☐
   - Decline to answer ☐

6. Do you have any pets at home?
   - Yes ☐
   - No ☐
   - Decline to answer ☐

7. Has your child **ever** had wheezing or whistling in the chest at any time in the past?
   - Yes ☐
     - o Has your child had wheezing or whistling in the chest in the **past 12 months?**
       - ▪ Yes ☐
       - ▪ No ☐
       - ▪ Don't know ☐
     - o How many attacks of wheezing has your child had in the **past 12 months?**
       - ▪ None ☐

        - 1 to 3 ☐
        - 4 to 12 ☐
        - More than 12 ☐
    - In the past 12 months, how often, on average, has your child's sleep been disturbed due to wheezing?
        - Never woken with wheezing ☐
        - Less than one night per week ☐
        - One or more nights per week ☐
- No ☐
- Don't know ☐

8. In the past 12 months, has your child's chest sounded wheezy during or after physical activity?
    - Yes ☐
    - No ☐
    - Don't know ☐

9. In the past 12 months, has your child had a dry cough at night, apart from a cough associated with a cold or chest infection?
    - Yes ☐
    - No ☐
    - Don't know ☐

10. Has a doctor or other health professional ever told your child that he/she has asthma?
    - Yes ☐
        - How old was he/she when he/she was first told that he/she has asthma? **two-digit year:____**
        - Does he/she still have asthma?
            - Yes ☐
            - No ☐
            - Don't know ☐
        - During the past 12 months, has he/she had an episode of asthma or an asthma attack?
            - Yes ☐
            - No ☐
            - Don't know ☐
        - During the past 12 months, has he/she had to visit an emergency room or urgent care center because of asthma?
            - Yes ☐
            - No ☐
            - Don't know ☐
        - During the past 3 months, has your child taken medication prescribed by a doctor or other health professionals for asthma?

        - Yes ☐
        - No ☐
        - Don't know ☐

    During the past week for how many days {child's name} could not attend school due to worsening of his/her asthma?

    NONE ☐
    1 DAY ☐
    2-3 DAYS ☐
    FULL WEEK ☐
    DON'T KNOW ☐

    - Did he/she develop asthma in Miami?
        - Yes ☐
        - No ☐
        - Don't know ☐
    - Once in Miami, his/her asthma symptoms have:
        - worsened ☐
        - improved ☐
        - not changed ☐
        - don't know
- No ☐
- Don't know ☐

11. Has a doctor or other health professional ever told your child that he/she has hay fever?
    - Yes ☐
        - How old was your child when he/she was first told he/she had hay fever? **two-digit year: ____**
        - During the past 12 months, has your child had an episode of hay fever?
            - Yes ☐
            - No ☐
            - Don't know ☐
        - Once in Miami, his/her episodes of hay fever have:
            - increased/occur more often ☐
            - decreased/occur less often ☐
            - not changed ☐
            - don't know
    - No ☐
    - Don't know ☐

12. Has a doctor or other health professional ever told your child that he/she has allergies?

- Yes ☐
  - How old was your child when he/she was first told he/she had allergies? **two-digit year: ____**
  - During the past 12 months, has your child had any allergy symptoms or an allergy attack?
    - Yes ☐
    - No ☐
    - Don't know ☐
  - Once you moved to Miami, his/her allergy symptoms have:
    - increased in frequency or severity ☐
    - decreased in frequency or severity ☐
    - not changed ☐
    - don't know ☐
- No ☐
- Don't Know ☐

13. Has your child been tested for "environmental allergies"?

- Yes ☐
  - At what age was he/she tested? ________
  - Where was he/she tested?
    - Florida ☐
    - Elsewhere ☐
      - **city/state:______** OR **country, if not in the U.S.: ___________**
  - Please indicate which allergens, if any, your child was found to be allergic to based on that test (please tick all that apply):
    - ☐ *Alternaria alternata* (a mold)
    - ☐ *Asperigillus fumigatus (a mold)*
    - ☐ Australian pine (*Causarina equisetifolia*)
    - ☐ Bahia grass (*Paspalum notatum*)
    - ☐ Bermuda grass (*Cynodon dactylon*)
    - ☐ *Blomia tropicalis* (a dust mite)
    - ☐ Cat dander
    - ☐ *Cladosporium herbarum (a plant fungus)*
    - ☐ Cockroach
    - ☐ Common ragweed (short; *Ambrosia elatior*)
    - ☐ *Dermatophagoides farinae (a house dust mite)*
    - ☐ *Dermatophagoides pteronyssinus* (a house dust mite)

- ☐ Dog dander
- ☐ Elm (*Ulmus americana*)
- ☐ Maple (box elder; *Acer negindo*)
- ☐ Mountain cedar (*Juniperus sabinoides*)
- ☐ Mouse Urine Proteins
- ☐ Nettle (*Urtica dioica*)
- ☐ Oak (*Quercus alba*)
- ☐ *Penicillin*
- ☐ Rough pigweed (*Amaranthus retroflexus*)
- ☐ Sheep sorrel (*Rumex acetosella*)
- ☐ Timothy grass (*Phleum pratense*)
- ☐ Other: please list _______________________
- ☐ None

- No ☐
- Don't know ☐

13.a During the past 30 days for how many days did {child's name} take anti-allergy medicines (e.g. Allegra, Zyrtec, Claritin, Benadryl)?

NOT A SINGLE DAY………………………1
1-2 DAYS……………………………………2
3-5 DAYS……………………………………3
5-10 DAYS…………………………………..4
10-30 DAYS…………………………………5
EVERY DAY………………………………...6
EVERY TWICE...…………………………...7

14. Do you know of any other allergies that your child has (not based on formal testing, but based on experience)?
    - Yes ☐
        - Please list these:__________________________
    - No ☐
    - Don't know ☐

15. In the last 12 months, has a dog, cat, or other small furry animal been removed from your child's home because your child had allergies or asthma?
    - Yes ☐
        - What kind of pet was removed from your home? __________
    - No ☐

16. Have you avoided bringing new pets into your home because your child had allergies or asthma?
    - Yes ☐
    - No ☐

17. Has your child **ever** had a problem with sneezing, or a runny, blocked nose when he/she did not have the cold or the flu?
    - Yes ☐
        - o At what age did this nose problem first occur? **two-digit year: ________**
        - o In the **past 12 months**, has your child had a problem with sneezing, or a runny, or blocked nose when he/she did not have the cold or the flu?
            - Yes ☐
            - No ☐
        - o In which of the past 12 months did this nose problem occur? (Please tick all tat apply)
            - January ☐
            - February ☐
            - March ☐
            - April ☐
            - May ☐
            - June ☐
            - July ☐
            - August ☐
            - September ☐
            - October ☐
            - November ☐
            - December ☐
        - o Once you moved to Miami, his/her nose symptoms have:
            - Increased in frequency or severity ☐
            - Increased in frequency or severity ☐
            - Not changed ☐
            - Don't know ☐
    - No ☐
    - Don't know ☐

18. Has a doctor or other health professional ever told your child that he/she has eczema?
    - Yes ☐
        - How old was your child when he/she was first told he/she has eczema? **two-digit year____**

- No ☐
- Don’t Know ☐

19. Has a doctor or other health professional ever told your child that he/she has eczema?
    - Yes ☐
        - How old was your child when he/she was first told he/she has eczema? **two-digit year____**
    - No ☐
    - Don’t Know ☐

Q20. Do you pay close attention to the outdoor pollen in your area?

NEVER ☐
RARELY ☐
SOMETIMES ☐
MOST OF THE TIME ☐
ALWAYS ☐

20.a During the past week, how often did you and/or any of the family members access pollen count or allergy index from pollen.com, weather.com or any other local/state/private agency?

NONE ☐
1 DAY ☐
2-3 DAYS ☐
EVERY DAY ☐
TWICE A DAY ☐

20b. How often do you change your air filter in your air conditioning system?

NEVER ☐
EVERY MONTH ☐
EVERY TWO MONTHS ☐
EVERY SIX MONTHS ☐
ONCE A YEAR ☐
DON’T KNOW ☐

20.c Do you have any germicidal air purification system, such as ultraviolet-C, in your air condition system?

YES …...………………………1
NO……..………………………. 2
DON’T KNOW………………..-99

20.d Do you use an air purifier (an electric machine used to clean air) in home?

YES …...………………………1
NO…….………………………. 2
DON'T KNOW………………..-99

20.e Do you ever feel moldy (or must smell) inside your home?

YES …...………………………1
NO…….………………………. 2
NOT SURE…………………..-99

20.f Can you regulate humidity through your air condition/heating system

YES ☐
NO ☐
DON'T KNOW ☐

Q20.g. During the last 12 months, were any areas inside your home painted, such as walls, trim or ceilings?

YES... 1
NO... 2
REFUSED.. 7
DON'T KNOW.. 9
